# Exploring thyroid eye disease in Brazil: insights from a single-center study

**DOI:** 10.20945/2359-4292-2024-0495

**Published:** 2025-08-18

**Authors:** Danilo Villagelin, Nicolas Perini, Roberto Bernardo Santos, João Hamilton Romaldini

**Affiliations:** 1 Pontifícia Universidade Católica de Campinas, Escola de Ciências da Vida, Faculdade de Medicina, Campinas, SP, Brasil; 2 Universidade Estadual de Campinas, Pós-graduação em Medicina Interna, Campinas, SP, Brasil; 3 Hospital da Pontifícia Universidade Católica de Campinas, Departamento de Endocrinologia, Campinas, SP, Brasil

**Keywords:** Thyroid eye disease, Brazil, Graves’, orbitopathy

## Abstract

**Objective:**

Graves’ disease (GD) is the leading cause of hyperthyroidism globally, with 40% of affected individuals developing thyroid eye disease (TED). Treatment options for TED have advanced in recent years. This study aimed to investigate the prevalence of TED at a single center in Brazil, contributing more robust data for cost-analysis studies assessing the financial implications of novel TED treatments. Subjects and

**methods:**

This study evaluated the clinical histories of 660 patients diagnosed with GD from 1999 to 2019. The patients were categorized into four groups based on the presence and severity of TED.

**Results:**

The prevalence of TED within the study population, categorized according to severity, was as follows: absent (n = 325; 49%), mild (n = 221; 33%), moderate to severe (n = 107; 16%), and sight-threatening (n = 7; 1%). A significant correlation was observed between older age at diagnosis, smoking, larger goiters, and the presence and severity of TED.

**Conclusion:**

The prevalence of TED identified in this single-center study contributes valuable insights for the design of cost-analysis studies and the assessment of the financial implications of novel treatments for TED within both the public and private healthcare systems in Brazil.

## INTRODUCTION

Graves’ disease (GD) is the leading cause of hyperthyroidism globally (^1^). This condition is characterized by an autoimmune thyroid disease caused by thyrotropin (TSH) receptor antibodies (TRAb), resulting in clinical thyrotoxicosis and extrathyroidal manifestations.

Thyroid eye disease (TED) is the most prevalent extrathyroidal manifestation of GD, affecting 40% of patients (^2^). In TED, TRAb binds to the TSH receptor in orbital fibroblasts, triggering an inflammatory response mediated by T lymphocytes and promoting a cellular response (^3^). This response results in the elevation of inflammatory cytokines within the orbital tissue, thereby exacerbating the inflammatory response and facilitating the accumulation of glycosaminoglycans and hyaluronic acid, which subsequently promotes localized edema (^3^). This autoimmune process leads to long-term clinical manifestations with significant morbidity and potential sight-threatening consequences (^4^).

In recent years, the treatment of TED has progressed from traditional approaches, such as glucocorticoids and radiotherapy, to a novel class of immunobiological agents. These agents encompass off-label therapies including rituximab (^5^) and tocilizumab (^6^), as well as treatments that have received approval by the United States Food and Drug Administration (FDA) and the Brazilian National Health Surveillance Agency (*Agência Nacional de Vigilância Sanitária* - Anvisa), notably teprotumumab (^7,8^). These novel pharmacological agents have shown efficacy in crucial endpoints associated with TED, such as reduction of inflammation, proptosis, and diplopia (^9^). However, they are associated with side effects and are more costly than glucocorticoids (^9^).

In Brazil, the regulatory procedure following Anvisa approval enables the integration of medications into public or private insurance systems. During the incorporation process, a stringent protocol is observed. Within the context of TED, it is essential to ascertain its prevalence in Brazil, as the cost analysis and financial implications are contingent upon these data in both scenarios.

The prevalence of TED in Europe and the United States is well documented and has been published for several decades. However, there is a lack of data concerning the prevalence of TED among patients with GD in Brazil. Based on this consideration, the aim of this study was to evaluate the prevalence of TED and its different presentations in a large Brazilian cohort of patients diagnosed with GD and compare this finding to those of previous studies in other geographical regions.

## SUBJECTS AND METHODS

This study assessed the clinical histories of patients diagnosed with GD at a single center from January 1999 to December 2019. Patients diagnosed after 2020 were intentionally excluded from the study to mitigate potential biases introduced by the COVID-19 pandemic, *i.e.*, cases of GD linked to the coronavirus (^10^), instances of vaccines associated with the development of TED (^11^), and delays in appointments resulting from the pandemic’s impact on the Brazilian healthcare system. Patients who developed TED in the absence of hyperthyroidism were also excluded from the analysis due to insufficient clinical history data.

The diagnostic criteria for GD included the presence of hyperthyroidism indicated by increased serum levels of free thyroxine (FT4) and reduced or suppressed serum levels of TSH, presence of a diffuse goiter identified through ultrasonography, and positive serum thyrotropin receptor antibody (TRAb) or increased uptake of ^99m^Tc-pertechnetate on thyroid scintigraphy. The diagnosis of TED was made by two authors (DV and RSB) using a Hertel exophthalmometer, evaluation of clinical activity score (^12^), classification according to the 2021 European Group on Graves’ Orbitopathy (EUGOGO) guidelines (^13^), and assessment of diplopia (^14^). All patients classified as having moderate-to-severe TED or higher according to the EUGOGO guidelines were referred to an ophthalmologist. Most of these patients underwent orbital imaging evaluation using either computed tomography or magnetic resonance imaging.

The patients’ data were obtained from their medical records. Since 2007, the institution has maintained an electronic medical record system with standardized documentation for patients with GD. All patients are evaluated for TED.

The patients were categorized into groups based on the presence and severity of their initial presentation of TED. The diagnosis of TED was concurrently established with the diagnosis of GD in most cases, whereas in fewer than 10% of the cases, the diagnosis was determined either prior to or subsequent to the identification of GD. The thyroid function of the patients, along with autoimmune biomarkers and clinical characteristics, were also assessed.

The study was approved by the institution’s ethics committee (CAAE: 39062520.8.0000.5481).

## RESULTS

The study included 660 patients. Their mean age was 42 ± 13 years, 80% were women, and 34% reported smoking.

Patients were categorized according to the severity of TED: absent in 49% (325/660) of patients, mild in 33% (221/660), moderate to severe in 16% (107/660), and sight-threatening in 1% (7/660).

Table 1 describes the clinical characteristics of the groups according to TED severity.

**Table 1:** Clinical characteristics of patients with thyroid eye disease, categorized according to disease severity

	Absent (n=325)	Mild (n = 221)	Moderate to severe (n = 107)	Sight-threatening (n = 7)	P value
Percentage of patients	49%	33%	16%	1%	
Age (years)a	42 (33-52)	39 (30-47)	39 (32-50.5)	41 (33.5-44)	<0.005
Female sex	80%	82%	73%	14%	<0.005
TSH (IU/mL)^a^	0.010 (0.005-0.010)	0.010 (0.005-0.010)	0.010 (0.005-0.010)	0.016 (0.006-0.042)	<0.005
FT4 (ng/dL)^a^	2.92 (1.94-5.12)	4.61 (2.66-6.00)	4.09 (2.24-5.47)	2.63 (2.34-5.20)	<0.005
Proptosis (mm)^a,b^	16 (14-18)	18 (16-20)	22 (18-24)	26 (23-26.5)	-
Eye aperture (mm)^a,b^	10 (8-10)	10 (10-12)	12 (11-14)	10 (10-10)	-
Thyroid volume on ultrasonography (mL)^a^	16.6 (12.0-28.3)	23.7 (15.9-39.3)	19.7 (13.5-34.6)	41.0 (31.5-50.5)	<0.005
Anti-TPO (positive)	70%	71%	70%	14%	<0.005
Anti-Tg (positive)	46%	43%	42%	14%	NS
TRAb (positive)	80%	81%	92%	100%	<0.005
Smoking (present)	31%	30%	54%	60%	<0.005

a Data are shown as median (25th-75th percentiles).

b Measured in the eye with the worst disease expression. P values were obtained using the Mann-Whitney test for comparisons of mean values and the chi-square test for comparisons of proportions. Abbreviations: Anti-TPO, antithyroid peroxidase antibody; Anti-Tg, antithyroglobulin antibody; FT4, free thyroxine; NS, non-significant; TRAb, thyrotropin receptor antibody; TSH, thyroid-stimulating hormone.

## DISCUSSION

The present study assessing the prevalence of TED in a large cohort of patients with GD found that it was absent in 49% (325/660) of patients, mild in 34% (221/660), moderate to severe in 16% (107/660), and sight-threatening in 1% (7/660).

The global prevalence of TED among patients with GD is reported to be 38% in Europe (^12^) and 44% (^15^) in the United States, whereas it is documented to be lower at 27% in Asia (^16^) and higher at 58% in Oceania (^17^). A recent meta-analysis investigating populations in Europe and North America has determined a prevalence of TED among patients with GD of approximately 40% (^18^), indicating a potential gradual increase in prevalence in recent years (^15^). Numerous factors contribute to the regional disparities in the prevalence of TED, including genetic predisposition, environmental influences, smoking status, and the characteristics of the healthcare system (^19,20^).

The findings of the present study provide important new insights into the prevalence of TED in Brazil, given the absence of existing data on this topic. According to the Brazilian National Committee for Health Technology Incorporation in the Unified Health System (*Comissão Nacional de Incorporação de Tecnologias no Sistema Único de Saúde* - Conitec), the incorporation process of a novel medication involves various studies, including cost-effectiveness analyses and assessment of financial impact. Consequently, national data are fundamental for the development of accurate reports (^21^).

The findings of this single-center cohort study suggest that the risk factors for TED observed in the Brazilian demographic are consistent with those reported in diverse populations (^14^). Specifically, the present study found a significant correlation between older age at diagnosis, smoking, larger goiters, and the presence and severity of TED, which aligns with results reported in the literature concerning European and American populations (^22,23^). In light of the inherent limitations associated with the design of this study, the findings suggest that age, tobacco use, and larger goiter may be potential risk factors for TED within the studied population.

The limitations of the present study include the analysis of a cohort derived from a referral center, which may have introduced bias into the results. This is due to the likelihood that the disease severity among the patients at this center is greater than that observed in the general population. Another limitation of the study is the lack of ophthalmological evaluation for patients categorized in the mild group, as such evaluations were not available at the time of GD diagnosis, and the study’s objective was to ascertain the prevalence of TED at the point of GD diagnosis.

In conclusion, the present study identified a prevalence of TED that exceeded that reported in other geographical locations, while also establishing an association with the same well-documented risk factors recognized in diverse regions worldwide. These findings contribute valuable insights and facilitate more accurate evaluations in cost-analysis studies focused on the integration of novel treatments for TED within both the public and private healthcare systems.
